# Cell cycle arrest in Metformin treated breast cancer cells involves activation of AMPK, downregulation of cyclin D1, and requires p27^Kip1 ^or p21^Cip1^

**DOI:** 10.1186/1750-2187-3-18

**Published:** 2008-12-01

**Authors:** Yongxian Zhuang, W Keith Miskimins

**Affiliations:** 1 Cancer Biology Research Center, Sanford Research/USD, 1400 West 22nd Street, Sioux Falls, South Dakota, 57105, USA; 2 Sanford School of Medicine of the University of South Dakota, Sioux Falls, SD 57197, USA

## Abstract

**Background:**

The antihyperglycemic drug metformin may have beneficial effects on the prevention and treatment of cancer. Metformin is known to activate AMP-activated protein kinase (AMPK). It has also been shown to inhibit cyclin D1 expression and proliferation of some cultured cancer cells. However, the mechanisms of action by which metformin mediates cell cycle arrest are not completely understood.

**Results:**

In this study, metformin was found to inhibit proliferation of most cultured breast cancer cell lines. This was independent of estrogen receptor, HER2, or p53 status. Inhibition of cell proliferation was associated with arrest within G0/G1 phase of the cell cycle. As in previous studies, metformin treatment led to activation of (AMPK) and downregulation of cyclin D1. However, these events were not sufficient for cell cycle arrest because they were also observed in the MDA-MB-231 cell line, which is not sensitive to growth arrest by metformin. In sensitive breast cancer lines, the reduction in cyclin D1 led to release of sequestered CDK inhibitors, p27^Kip1 ^and p21^Cip1^, and association of these inhibitors with cyclin E/CDK2 complexes. The metformin-resistant cell line MDA-MB-231 expresses significantly lower levels of p27^Kip1 ^and p21^Cip1 ^than the metformin-sensitive cell line, MCF7. When p27^Kip1 ^or p21^Cip1 ^were overexpressed in MDA-MB-231, the cells became sensitive to cell cycle arrest in response to metformin.

**Conclusion:**

Cell cycle arrest in response to metformin requires CDK inhibitors in addition to AMPK activation and cyclin D1 downregulation. This is of interest because many cancers are associated with loss or downregulation of CDK inhibitors and the results may be relevant to the development of anti-tumor reagents that target the AMPK pathway.

## Background

Metformin hydrochloride (N, N-dimethylimidodicarbonimidic diamide hydrochloride) is a commonly prescribed oral antihyperglycemic drug used in the management of Type 2 diabetes. Recent evidence indicates that metformin has significant effects on tumorigenesis and cancer cell growth. It was reported that patients with Type 2 diabetes who are prescribed metformin have a lower risk of cancer compared to patients that do not take metformin [1,2]. In a mouse xenograft model, metformin suppressed tumor growth of p53 negative HCT116 colon cancer cells, but not of p53 wild-type cells [3]. Metformin treatment decreases the incidence and size of mammary adenocarcinomas in Her2/Neu mice [4] and prevents carcinogen-induced pancreatic cancer in hamsters [5]. In culture, metformin has been shown to inhibit growth of cells derived from breast cancer, colon cancer, prostate cancer, and gliomas [3,6-9]. However, the mechanisms of action by which metformin mediates these beneficial effects on cancer cell growth are not well understood.

One intracellular target of metformin is the activation of adenosine 5'-monophosphate-activated kinase (AMPK) [10]. AMPK consists of three subunits, α,β and γ, and each subunit has at least two isoforms [11]. Activation of AMPK involves AMP binding to regulatory sites on the γ subunits. This causes conformational changes that allosterically activate the enzyme and inhibit dephosphorylation of Thr172 within the activation loop of the catalytic α subunit [12,13]. LKB1 has been identified as an upstream kinase and shown to phosphorylate the α subunit of AMPK at Thr172 [14-16]. However, metformin most likely does not directly activate either LKB1 or AMPK and the drug does not influence the phosphorylation of AMPK by LKB1 *in vitro *[14,17,18]. Rather, there is evidence that AMPK activation in response to metformin treatment is downstream of effects on complex 1 of the mitochondrial electron transport chain [19-22].

It is interesting to note that LKB1 is a well recognized tumor suppressor and mutations in the gene encoding LKB1 cause the rare inherited Peutz-Jeghers syndrome [23]. It is believed that the LKB1-AMPK pathway functions as a cellular energy-sensing checkpoint that controls cell growth and proliferation according to the availability of fuel supplies [24]. Considering the important role of the LKB1-AMPK pathway in cell growth control, it is a potential target for cancer treatment or prevention. Metformin stimulates this pathway and modulates tumor cell growth, *in vitro *and *in vivo*, but its mode of action remains unclear. In this report we demonstrate that metformin-sensitive breast cancer cells arrest in G0/G1 due to activation of AMPK, downregulation of cyclin D1, and enhanced binding of CDK2 by p27^Kip1 ^and p21^Cip1^. AMPK is activated and cyclin D1 is downregulated in a metformin-resistant breast cancer cell line. However, this cell line becomes sensitive to metformin when p27^Kip1 ^or p21^Cip1 ^is overexpressed. Thus, CDK inhibitors are essential for cell cycle arrest in response to metformin. This is of significance because p27^Kip1 ^is often downregulated in cancer cells.

## Results

### Metformin treatment has cell line-dependent effects on breast cancer cells

**Figure 1:**
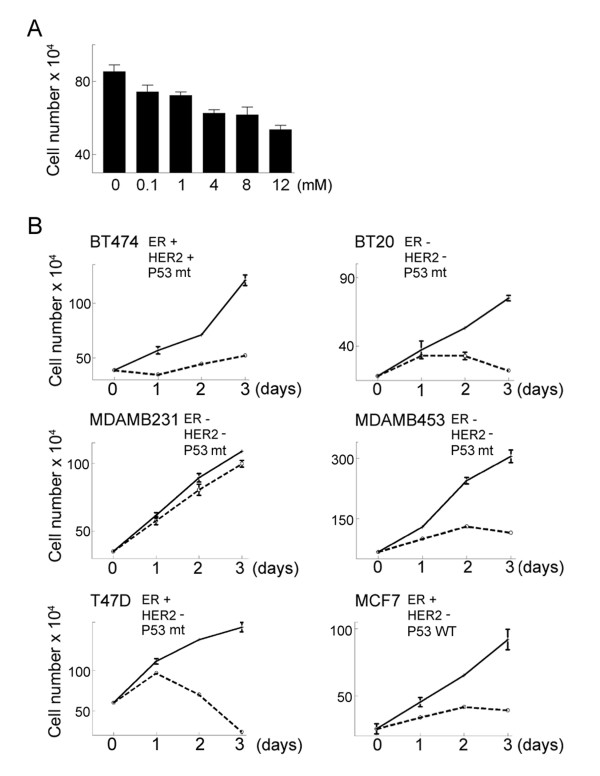
**Metformin inhibits proliferation of cultured breast cancer cells**. **A**. MCF7 cells were treated with metformin at the indicated concentrations for one day and then cell number was determined using a hemocytometer. The mean cell number from 3 independent cultures is shown. Error bars represent standard deviation. Using a t test, all treatments were significantly different from the control with P values of less than 0.01. **B**. Six different breast cancer cell lines were treated with 8 mM metformin. At the indicated times viable cell number was determined using a hemocytometer. The reported status of estrogen receptor (ER) expression, HER2 amplification, and p53 expression (Wild-type, WT, or mutant, mt) are indicated for each cell line. HER2+ indicates HER2 amplification while HER2- indicates normal HER2 expression. The data points represent the mean cell number from 3 independent cultures and error bars represent standard deviation.

While exploring the potential role of AMPK in regulating p27^Kip1 ^expression, we noted that metformin, an AMPK activator, inhibits proliferation of MCF7 cells in a dose dependent manner (Fig. 1A). We further explored the effects of metformin on proliferation by treating a panel of breast cancer cell lines with the drug at a concentration of 8 mM. As shown by the work of Owen et al[21], this represents a physiologically relevant dose of metformin. Five of six breast cancer cell lines, including MCF7, BT20, T47D, MDA-MB-453, and MDA-MB-474, underwent growth arrest after metformin treatment (Fig. 1B). However, the cell line MDA-MB-231 was resistant to the effects of metformin, showing only a slight decrease in proliferation over a four day period (Fig. 1B). These data indicate that metformin has differential effects on cell proliferation in a breast cancer cell line-dependent manner. Furthermore, there is no correlation between sensitivity to metformin and the known status of estrogen receptor (ER) expression, p53 mutation, or amplification of Her-2[25,26].

### Effects of metformin on cell cycle regulatory proteins in MCF7 cells

**Figure 2:**
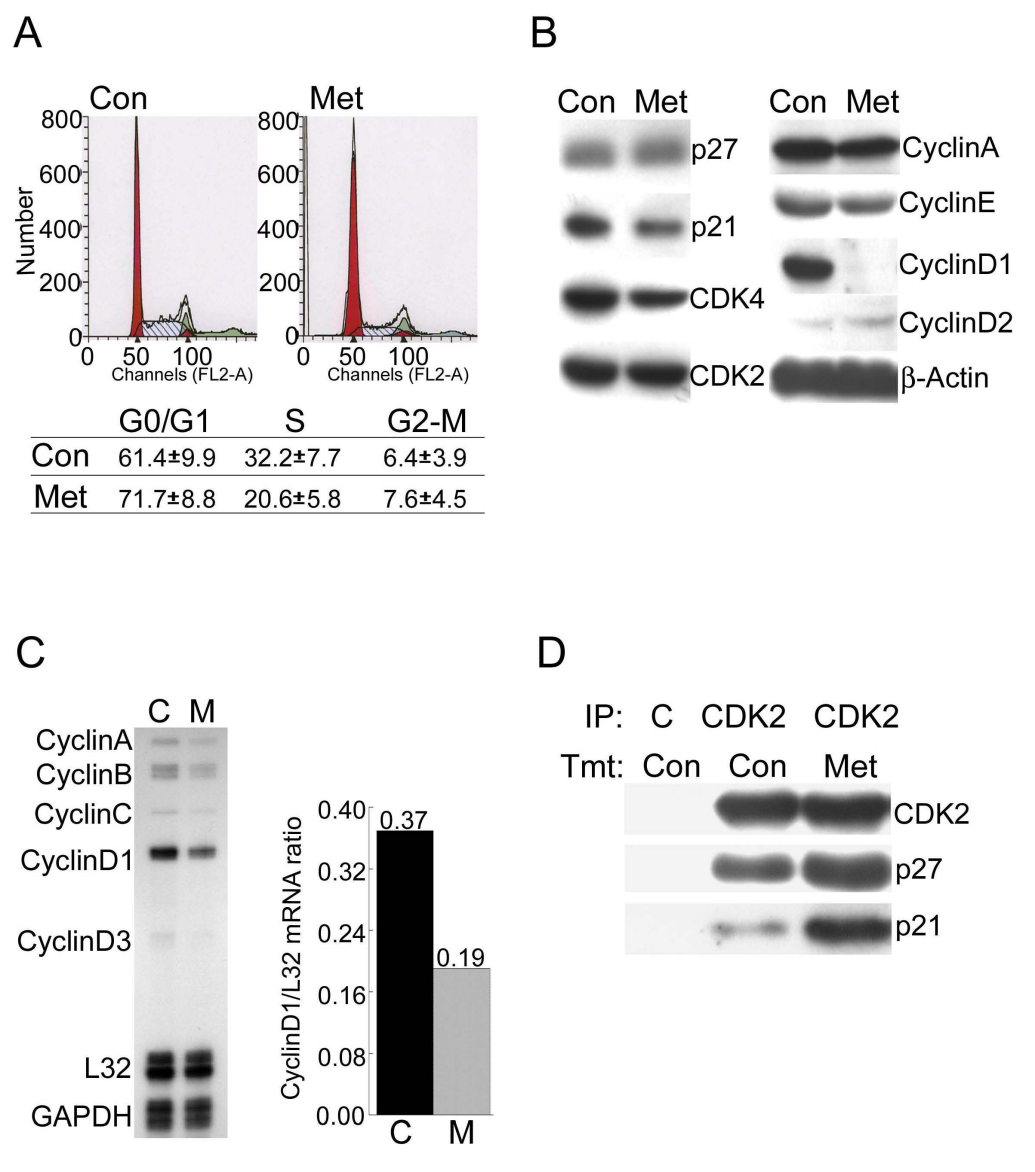
**Effect of metformin on cell cycle progression and cell cycle regulatory proteins in MCF7 cells**. **A**. Untreated cells (Con) or cells treated with 8 mM metformin for 1.5 days (Met) were stained with propidium iodide and then analyzed by flow cytometry to estimate the number of cells in each phase of the cell cycle. The experiment was repeated 3 times and the mean and standard error for each cell phase is indicated in the table. **B**. Equal amounts of protein from untreated cells (Con) or cells treated with metformin for 1.5 days (Met) were analyzed by western blotting using antibodies that recognize the indicated cell cycle regulatory proteins. β-Actin was detected as a loading control **C**. Total RNA was isolated from untreated cells (C) or cells treated with metformin (8 mM) for 1.5 days (M) and RNase protection assays were performed to detect the mRNAs encoding the indicated cyclins (left panel). Cyclin D1 mRNA levels were normalized using L32 mRNA levels (right panel). The mean of 3 independent experiments is shown and error bars indicate standard deviation. In a t test the value for the metformin treated cells was significantly different from the control with a P value of 0.006. **D**. MCF7 cells were treated with (Met) or without (Con) metformin (8 mM) and then extracts were prepared for immunoprecipitation (IP) using either a control antibody (C) or an anti-CDK2 antibody (CDK2). The immunoprecipitated proteins were analyzed by western blotting using antibodies that recognize CDK2, p27^Kip1 ^or p21^Cip1 ^as indicated on the right.

Since MCF7 cells were sensitive to metformin-mediated inhibition of cell proliferation, we chose this cell line for further experiments to characterize the molecular mechanisms by which metformin arrests the cell cycle. In order to determine the effects of metformin on cell cycle progression, MCF7 cells were treated with the drug for 1.5 days and then flow cytometry was performed on propidium iodide stained cells (Fig. 2A). Metformin treatment increased cells in G0/G1 phase from 61.4% to 71.7% and decreased the number of cells in S phase from 32.2.54% to 20.6%. These data suggest that metformin inhibits cell cycle progression from G0/G1 into S phase. Therefore, western blotting was used to examine the effects of metformin on various cell cycle regulatory proteins, including cyclin E, cyclin D1, cyclin D2, cyclin A, CDK2, CDK4 and the CDK inhibitors p27^Kip1 ^and p21^Cip1 ^(Fig. 2B). The most remarkable change was the loss of cyclin D1 protein after metformin treatment. The effects of metformin on mRNA levels for several cell cycle regulatory proteins were also examined. MCF7 cells were treated with or without metformin for 1.5 days and then a multi-probe ribonuclease protection assay (RPA) was performed. Cyclin D1 mRNA levels were notably lower in metformin treated cells (Fig. 2C). When normalized to the house-keeping gene L32, there was a 36.1% reduction of cyclin D1 mRNA after metformin treatment. These data indicate that metformin-mediated downregulation of cyclin D1 is primarily due to decreased levels of cyclin D1 mRNA.

One function of the cyclin D1/CDK complex is the binding and sequestration of p27^Kip1 ^and p21^Cip1^. This prevents these proteins from binding to and inhibiting the cyclin E/CDK2 complex which promotes progression from G0/G1 to S phase of the cell cycle[27] Since metformin causes downregulation of cyclin D1, this may lead to release of p27^Kip1 ^and p21^Cip1^, allowing them to bind to CDK2 and block its activity. To test this, co-immunoprecipitation was used to monitor the levels of p27^Kip1 ^and p21^Cip1 ^associated with CDK2 in cells treated with or without metformin. Immunoprecipitation was performed using anti-CDK2 antibody as well as an unrelated control antibody of the same isotype. Then western blotting was used to detect co-immunoprecipitated p27^Kip1 ^and p21^Cip1 ^(Fig. 2D). CDK2 levels were similar in anti-CDK2 immunoprecipitates from metformin treated and untreated cells. However the levels of p27^Kip1 ^and p21^Cip1 ^that co-immunoprecipitated with CDK2 were considerably elevated in metformin treated cells. These results support the conclusion that metformin blocks MCF7 cells from proliferating by decreasing cyclin D1 protein levels leading to release of sequestered p27^Kip1 ^and p21^Cip1^. The released CDK inhibitors subsequently bind to and inhibit cyclin E/CDK2 complex activity, leading to arrest in G0/G1.

### Downregulation of cyclin D1 involves activation of AMPK

**Figure 3:**
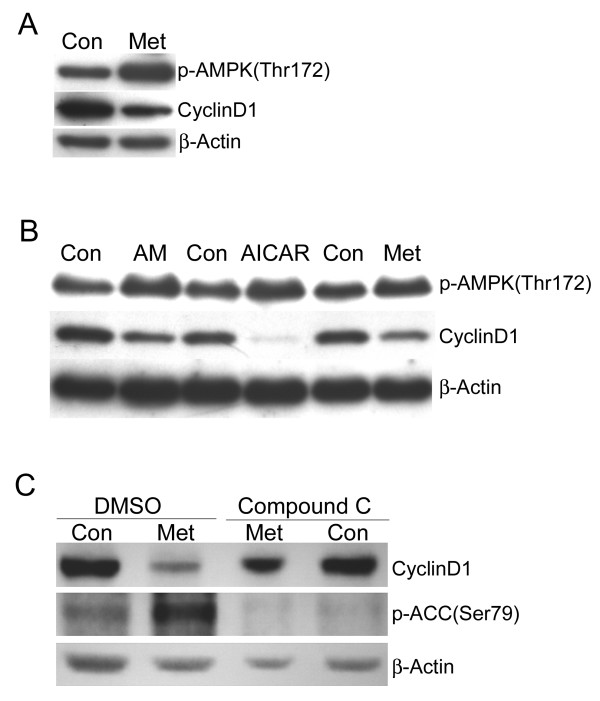
**Downregulation of cyclin D1 corresponds to activation of AMP-activated protein kinase**. **A**. MCF7 cells were treated with (Met) or without (Con) metformin for 1.5 days and then western blotting was performed to detect active phospho-AMPK, cyclin D1, and β-actin. **B**. MCF7 cells were treated with antimycin A (AM, 1 μM), AICAR (4 mM), or metformin (8 mM) for 1.5 days. Control cells (Con) were treated with the appropriate vehicle for each reagent. Western blotting was performed to detect active phospho-AMPK, cyclin D1, and β-actin. **C**. MCF7 cells were pretreated with DMSO (vehicle) or the AMPK-specific inhibitor compound C (20 μM) for 1 day and then treated with (Met) or without (Con) metformin for 1.5 days. Western blotting was performed to detect cyclin D1 or the phosphorylated form of the AMPK substrate ACC. β-actin was used as a loading control.

Metformin may affect a variety of cellular pathways but some of its effects are known to be due to enhanced activity of AMPK. It was therefore of interest to determine if AMPK is activated in metformin treated cells and if this is required for downregulation of cyclin D1. MCF7 cells were treated with metformin for 1.5 days and then western blotting was used to examine the levels of the active phosphorylated form of AMPK (phospho-Thr172). The results show that metformin enhances phospho-AMPK levels and that this correlates with downregulation of cyclin D1 (Fig. 3A). Other reagents that are known to activate AMPK, including antimycin A (1 μM) and AICAR (4 mM), were also tested. Both reagents also led to increased AMPK phosphorylation and decreased cyclin D1 protein levels (Fig. 3B). To further examine this, MCF7 cells were pretreated with the AMPK-specific inhibitor compound C (20 μM) or DMSO (vehicle) for 1 day and then treated with or without metformin for an additional 1.5 days. Cells were harvested and western blotting was used to examine cyclin D1 and phosphorylated ACC, a substrate of AMPK. Pretreatment with compound C blocks the reduction of cyclin D1 caused by metformin treatment (Fig. 3C). This corresponds to loss of ACC phosphorylation. These results indicate that downregulation of cyclin D1 by metformin is through an AMPK-dependent pathway.

### Stable overexpression of p27^Kip1 ^in MDA-MB-231 cells leads to metformin sensitivity

**Figure 4:**
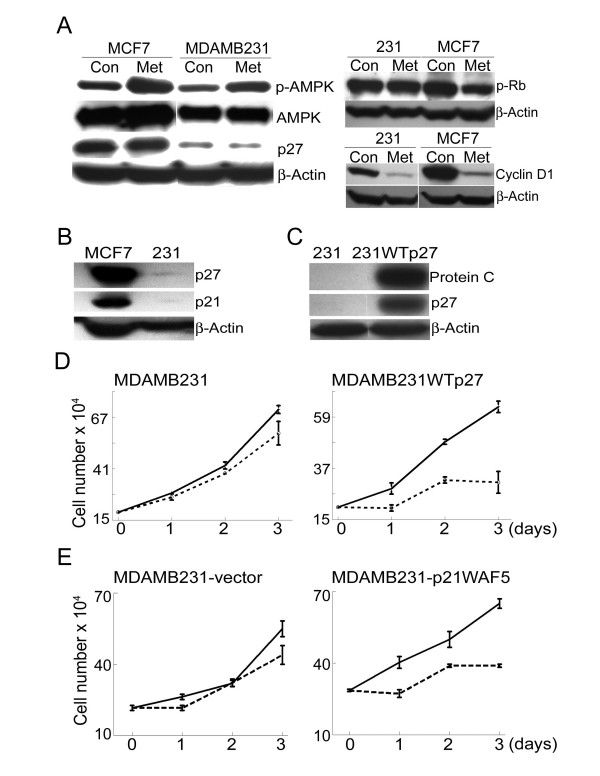
**Overexpression of p27^Kip1 ^in the metformin-resistant MDA-MB-231 cell line leads to metformin sensitivity**. **A**. (Left panel) MCF7 and MDA-MB-231 cells were treated with (Met) and without (Con) metformin (8 mM) for 2 days. Western blotting was used to detect phospho-AMPK, total AMPK, p27^Kip1^, and β-actin. (Right panel) Same as A except that cell extracts were used to detect phospho-Rb (Serine 795), cyclin D1 or β-actin. **B**. Extracts from untreated MCF7 and MDA-MB-231 cells were used for western blotting of p27^Kip1^, p21^Cip1^, and β-actin. **C**. MDA-MB-231 cells were stably transfected with a construct encoding p27^Kip1 ^that was tagged with a protein C epitope to derive the cell line MDA-MB-231WTp27. Extracts from the parental cell line (231) and the stably transfected cell line (231WTp27) were used for western blotting to detect the epitope-tagged p27^Kip1 ^(anti-Protein C epitope), p27^Kip1 ^(p27), and β-actin. **D**. MDA-MB-231 cells or MDA-MB-231WTp27 cells were treated with (dashed lines) or without (solid lines) metformin (8 mM) for up to 3 days. At the indicated time points cell number was determined using a hemocytometer. Each data point represents the mean cell number from 3 independent cultures and error bars represent standard deviation. **E**. MDA-MB-231 cells were transiently transfected with a construct encoding p21^Cip1 ^or empty vector. They were then treated with metformin for the indicated time and cell number was determined as describe in D.

MDA-MB-231 is an aggressive breast cancer cell line that does not express estrogen receptors and has a mutated form of p53 [25]. Our data show that metformin treatment does not prevent cell proliferation in this cell line (see Fig. 1). The lack of response to metformin treatment in MDA-MB-231 cells provided us an opportunity to test whether inhibition of cell proliferation by metformin is dependent on cyclin D1 downregulation followed by enhanced binding of the cyclin E/CDK2 complex by p27^Kip1 ^and p21^Cip1^. As indicated by an increased level of the phosphorylated enzyme, metformin treatment increased phospho-AMPK in MDA-MB-231 cells, similar to that observed in MCF7 cells (Fig. 4A). This indicates that MDA-MB-231 is responding to the drug. Also, cyclin D1 was decreased in MDA-MB-231 cells (Fig. 4A). MDA-MB-231 cells express p27^Kip1 ^and p21^Cip1 ^proteins at greatly reduced levels compared to MCF7 cells (Fig. 4B). This suggested the possibility that the resistance of MDA-MB-231 cells is due to low levels of these CDK inhibitors. Thus, even though cyclin D1 is downregulated in response to metformin treatment, there are insufficient levels of sequestered p27^Kip1 ^and p21^Cip1 ^to inhibit the activity of cyclin E/CDK2. This is supported by the finding that phosphorylation of Rb at serine 795, a site targeted by CDK2[28], is decreased in MCF7 cells but not in MDA-MB-231 cells following metformin treatment (Fig. 4A). In order to further test this hypothesis, we created a stable cell line, MDA-MB-231-WTp27, which overexpresses wild type p27^Kip1 ^(Fig. 4C). Compared to MDA-MB-231 cells, this stable cell line has a much higher level of p27^Kip1^. Metformin treatment of MDA-MB-231-WTp27 cells strongly inhibited proliferation (Fig. 4D). We have been unsuccessful in establishing MDA-MB-231 cells that stably overexpress p21^Cip1^. However, transient transfection of this line with an expression construct encoding p21^Cip1 ^also caused them to become more sensitive to growth inhibition in response to metformin (Fig. 4E). There was no difference in cell death between the metformin treated and untreated cultures as indicated by floating cells or trypan blue staining (data not shown). These results support the conclusion that downregulation of cyclin D1, alone, is not sufficient to arrest cells in response to metformin. CDK inhibitors p27^Kip1 ^or p21^Cip1 ^must be expressed at sufficient levels such that their release and subsequent inhibition of cyclin E/CDK2 leads to cell cycle arrest.

## Discussion

Several recent publications indicate that metformin has inhibitory effects on tumor cell growth *in vitro *and *in vivo *[1-9] The mechanisms through which metformin affects cell cycle progression and tumor growth have not been fully defined. The novel findings presented here are 1) that metformin inhibits the proliferation of most cultured breast cancer cell lines and that this does not correlate with the status of ER, Her-2, or p53 expression; 2) cyclin D1 levels are sharply downregulated in response to metformin but this alone is not sufficient to cause cell cycle arrest; and 3) cell cycle arrest also requires sufficient levels of CDK inhibitors (i.e. p27^Kip1 ^or p21^Cip1^) to bind and inhibit CDK2.

Downregulation of cyclin D1 in response to metformin has been demonstrated in LNCaP prostate cancer cells [8] but the importance of CDK inhibitors was not addressed in this study. These investigators did observe an small increase in p27^Kip1 ^levels following metformin treatment of LNCaP cells. We have not observed significant changes in the levels of CDK inhibitors in breast cancer cells. Downregulation of cyclin D1 protein in response to metformin correlated with decreased cyclin D1 mRNA levels. This suggests that metformin could initiate signaling pathways that lead to repression of the cyclin D1 gene. However, we have been unable to show any effect of metformin on the human cyclin D1 promoter (-1063) in luciferase reporter assays (data not shown). It is possible that metformin targets elements outside of the 5'-flanking region we have used for our experiments or that it causes destabilization of the cyclin D1 mRNA.

In the studies performed by Sahra et al[8] using LNCaP prostate cancer cells, inhibition of AMPK expression using siRNAs did not prevent metformin-induced downregulation of cyclin D1 or G0/G1 phase arrest. This is in contrast with our results showing that compound C, a specific inhibitor of AMPK, blocks metformin-induced downregulation of cyclin D1 in MCF7 cells. Zakikhani et al[9] have also shown that siRNAs targeting AMPK prevent growth inhibition of MCF7 cells in response to metformin treatment. The reasons for the differences are not known but could be related to the different cell types and their specific genetic backgrounds.

The cell line MDA-MB-231 is resistant to the growth inhibitory effects of metformin. It was previously reported that this cell line does not express the AMPK kinase LKB1[29,30]. It was also shown that MDA-MB-231 cells do not respond to metformin in terms of inhibition of protein translation through the mTOR pathway[29]. Our data show that the active phosphorylated form of AMPK increases in MDA-MB-231 cells in response to metformin. We also show that cyclin D1 is downregulated in metformin treated MDA-MB-231. Thus MDA-MB-231 cells are capable of responding to metformin and this could be through AMPK kinases other than LKB1. Both calmodulin-dependent protein kinase kinase [31-33] and ATM have been shown to phosphorylate and activate AMPK[34,35]. Further examination of these pathways will be needed to determine if they play a role in AMPK activation in response to metformin.

Even though MDA-MB-231 cells respond to metformin in terms of AMPK phosphorylation and cyclin D1 downregulation, these responses are not sufficient for cell cycle arrest. This cell line expresses very low levels of p27^Kip1 ^and p21^Cip1^. When p27^Kip1 ^is overexpressed, these cells stop proliferating following metformin treatment. We also found that overexpression of p21^Cip1 ^sensitized the cells to cell cycle arrest in response to metformin, indicating that either CDK2 inhibitor is capable of mediating this effect. Thus, CDK inhibitors are important mediators of the observed cell cycle arrest and appear to act downstream of AMPK activation and cyclin D1 loss. Other AMPK activators such as AICAR and antimycin A also caused downregulation of cyclin D1. A previous report showed that the proliferation of metformin-resistant MDA-MB-231 cells is inhibited by AICAR[36]. Thus, even though both reagents stimulate AMPK activity, the cellular responses to metformin and AICAR are not the same in MDA-MB-231 cells. AICAR is thought to activate AMPK by mimicking AMP, but it has been shown to have AMPK-independent effects on cells[37,38]. For metformin, it is thought that the primary target is complex I of the mitochondrial respiratory chain and that this is upstream of AMPK activation [19-22].

Using mouse embryo fibroblasts, Jones et al[39] showed that activation of AMPK induces phosphorylation of p53 and that this is required for AMPK-dependent cell cycle arrest. The same group showed, using paired isogenic colon cancer cell lines, that metformin selectively inhibited p53 negative tumor cell growth *in vivo*[3] With the panel of cultured breast cancer cell lines used in our experiments, we have not observed any correlation between either cell cycle arrest or cell survival and p53 expression. For example, of the cell lines shown in Fig. 1, all but MCF7 have been reported to carry p53 mutations[26], yet only MDA-MB-231 is resistant to the growth inhibitory effects of metformin in culture.

## Conclusion

**Figure 5:**
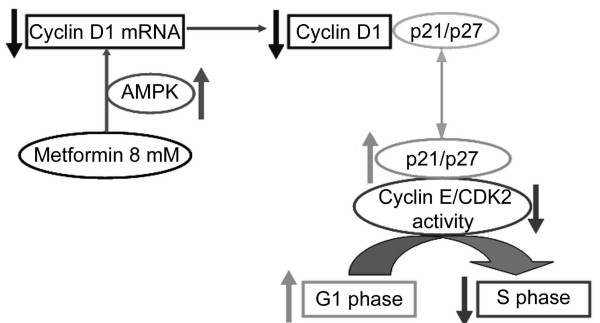
**Proposed model for the mechanism by which metformin mediates cell cycle arrest**. Metformin treatment leads to activation of AMPK which leads to loss of cyclin D1 mRNA and downregulation of cyclin D1 protein. The reduction in cyclin D1 results in the release of sequestered cell cycle inhibitors p27^Kip1 ^and p21^Cip1^. The released CDK inhibitors bind to and inhibit cyclin E/CDK2, thus preventing cell cycle progression from G1 to S phase. In metformin-resistant MDA-MB-231 cells, levels of the CDK inhibitors are insufficient to block CDK2 even though AMPK is active and cyclin D1 is downregulated.

Our data provide a molecular mechanism that may contribute to the ability of metformin to have beneficial effects on preventing or treating cancer. Our model (Fig. 5) is that metformin arrests cell proliferation by activating AMPK. Active AMPK leads to loss of cyclin D1 mRNA and protein. The decline in cyclin D1 levels causes the release of sequestered CDK inhibitors, p27^Kip1 ^and p21^Cip1^, which then bind to and inhibit the cyclin E/CDK2 complex. This prevents progression from G1 into S phase and blocks cell proliferation. Resistance to metformin mediated cell cycle arrest is observed when cells express low levels of p27^Kip1 ^or p21^Cip1^. Loss of p27^Kip1 ^expression is commonly observed in many types of cancer and is often correlated with poor prognosis. Therefore our findings may be of significance for development and use of anti-tumor drugs, such a metformin, that target the AMPK signaling pathway.

## Methods

### Chemicals

The following chemicals were used in this study: metformin (1, 1-dimethylbiguanide, Sigma Chemical Co), AICAR (5-aminoimidazole-4-carboxamide-riboside, Sigma Chemical Co.), AMPK inhibitor (Compound C, Calbiochem), antimycin A (Sigma Chemical Co.).

### Cell culture

All human breast cancer cell lines were purchased from ATCC and maintained in Dulbecco's modified Eagle's medium (DMEM) with 10% fetal bovine serum supplemented with 100 U/ml penicillin and 100 μg/ml streptomycin in a humidified CO2 incubator.

### Cell proliferation assay

Human breast cancer cells were plated into 35 mm dishes. After one day cells were treated with metformin at the indicated concentrations or the same volume of sterilized water. After incubation with metformin for 1, 2 or 3 days, cells were extensively rinsed in Dulbecco's phosphate buffered saline (PBS) to remove any loosely attached or floating cells. The cells were then harvested by trypsinization and cell number was determined using a hemocytometer.

### Western blotting

Cells in 35 mm dishes were rinsed with PBS and lysed by addition of sodium dodecylsulfate (SDS) sample buffer [2.5 mM Tris-HCl (pH 6.8), 2.5% SDS, 100 mM dithiothreitol, 10% glycerol, 0.025% pyronine Y]. Equal protein amounts were separated on 10% SDS-polyacrylamide gels. Proteins were transferred to Immobilon P membranes (Millipore) using a Bio-Rad Trans-blot apparatus with a transfer buffer of 48 mM Tris-HCl and 39 mM glycine. The membranes were blocked with 5% non-fat dry milk in Tris-buffered saline [10 mM Tris-HCl (pH 7.5), 150 mM NaCl] containing 0.1% Tween-20 (TBS-T) for 15–60 minutes at room temperature. The membrane was then incubated with the appropriate antibody in TBS-T containing 5% non-fat dry milk for 1 hour at room temperature or overnight at 4°C. After extensive washing in TBS-T the membrane was incubated with the appropriate horseradish peroxidase (HRP)-conjugated secondary antibody. After extensive washing in TBS-T, proteins were detected using the Super Signal West Pico chemiluminescent substrate (Pierce Biochemical). Anti-β-actin monoclonal antibody (A5441, used at 1:10,000) was purchased from Sigma. Antibodies against cyclin D1 (sc-753, used at 1:2500), cyclin D2 (sc-452, used at 1:2500), cyclin A (sc-751, used at 1:2500), cyclin E (sc-481, used at 1:2500), CDK2 (sc-163, used at 1:2500), CDK4 (sc-260, used at 1:2500), and p21^Cip1 ^(sc-817, used at 1:1000) were purchased from Santa Cruz Biotechnology. p27^Kip1^(610241, used at 1:2500) antibody was purchased from BD Biosciences. Antibodies against phosphorylated AMPK phosphorylated at threonine 172 (2535, used at 1:1000), AMPK (2532, used at 1:1000), and phospho-ACC (3661, used at 1:1000) were purchased from Cell Signaling Technology. Anti-protein C (1814508, used at 1:500) was purchased from Roche Applied Science. Secondary horseradish peroxidase-linked anti-mouse (31430, used at 1:5000) and anti-rabbit (31460, used at 1:5000) IgG antibodies were purchased from Pierce Biochemical.

### Immunoprecipitation

Cell extracts were prepared using a lysis buffer consisting of 50 mM HEPES, (pH 7.5), 150 mM NaCl, 1 mM EDTA, 2.5 mM EGTA, 10% glycerol, 0.1% Tween-20, 1 mM DTT, 1 mM NaF, 0.1 mM Na_3_VO_4_, 10 μg/ml leupeptin, 2 μg/ml Aprotinin, and 0.1 mM PMSF. Extracts were briefly sonicated and then centrifuged at 14,000 rpm in a MicroCL17R refrigerated microcentrifuge at 4°C for 15 min. Protein concentration was determined using the Bio-Rad protein assay. Equal amounts of total protein from each extract (0.5 to 1 mg) were used for analysis. Cell lysates were incubated with 2 μg of CDK2 antibody overnight at 4°C on a rotator. Protein A-conjugated agarose beads (30 μl) were added, and the incubation continued for 1 hour on a rotator. The beads were pelleted at 4,000 rpm using MicroCL17R refrigerated microcentrifuge at 4°C, the supernatant was discarded and the beads were washed five times in 500 μl lysis buffer. The precipitated proteins were dissolved in SDS-sample buffer, separated by SDS-PAGE and then p27^Kip1^, p21^Cip1 ^and CDK2 were detected by western blotting.

### Ribonuclease protection assay (RPA)

RPAs were carried out with the RiboQuant Multi-Probe RNase Protection Assay System (BD Biosciences). Human probe set hCYC-1 includes templates for cyclin A, cyclin B, cyclin C, cyclin D1, cyclin D2, cyclin D3, and cyclin A1, as well as for ribosomal protein L32 and GAPDH as controls. Probes were synthesized at room temperature using ^32^P-UTP and T7 RNA polymerase. Total cellular RNA was isolated using TRI Reagent (Molecular Research Center). The labeled riboprobe set was hybridized to 15 μg of RNA at 56°C for 12–16 h. After hybridization, free riboprobes and single-stranded RNA were digested with an RNase A and RNase T1 mixture at 30°C for 45 min. This was followed by treatment with proteinase K at 37°C. The remaining protected RNA fragments were purified by phenol-chloroform-isoamyl alcohol extraction followed by ethanol precipitation. The pellet was dried, dissolved in 5 μl loading buffer, heated at 90°C for 3 minutes and then placed in an ice bath. The sample was separated using a 4.75% denaturing polyacrylamide gel. The undigested labeled probe set was used as a marker. After electrophoresis RNase-protected bands were visualized by autoradiography using Kodak Biomax XAR film. The quantity of each mRNA species was estimated using a ChemiImager system.

### Transfection and selection of stable cell lines

MDA-MB-231 cells in 35 mm dishes were transfected with the construct pcDNA3.1-p27-protein C using Lipofectamine reagent (Invitrogen). After one day the cells were plated into 100 mm dishes and then selection with hygromycin (200 μg/ml) was initiated two days later. New medium with hygromycin was added every three days. After three weeks of selection, colonies were picked, expanded, and then tested by western blotting using anti-protein C and anti-p27 antibodies.

### Flow cytometry

MCF7 cells were treated with or without metformin for 1.5 days. Cells (1 × 10^6^) were trypsinized and fixed in 70% ethanol overnight. Fixed cells were stained with propidium iodide (50 μg/ml) for 30 minutes at room temperature. Cells were filtered using a 5 ml polystyrene round-bottom tube with a cell-strainer cap (BD Falcon) prior to flow cytometry. All flow cytometry measurements were done using a FACSVantage SE cell sorter (Becton Dickinson). Cell cycle analysis was performed using ModFit LT software.

## Competing interests

The authors declare that they have no competing interests.

## Authors' contributions

YZ carried out the all experimental procedures, participated in the design of the study, and assisted with manuscript preparation. WKM participated in the design and coordination of the study and helped to draft the manuscript. All authors read and approved the final manuscript.
